# Assessing the Congruence of Thermal Niche Estimations Derived from Distribution and Physiological Data. A Test Using Diving Beetles

**DOI:** 10.1371/journal.pone.0048163

**Published:** 2012-10-25

**Authors:** David Sánchez-Fernández, Pedro Aragón, David T. Bilton, Jorge M. Lobo

**Affiliations:** 1 Departamento de Departamento de Biogeografía y Cambio Global, Museo Nacional de Ciencias Naturales (CSIC), Madrid, Spain; 2 Departamento de Ecología e Hidrología, Universidad de Murcia, Murcia, Spain; 3 Institut de Biologia Evolutiva (CSIC-UPF), Barcelona, Spain; 4 Marine Biology and Ecology Research Centre, University of Plymouth, Plymouth, United Kingdom; CNRS/Université Joseph-Fourier, France

## Abstract

A basic aim of ecology is to understand the determinants of organismal distribution, the niche concept and species distribution models providing key frameworks to approach the problem. As temperature is one of the most important factors affecting species distribution, the estimation of thermal limits is crucially important for inferring range constraints. It is expectable that thermal physiology data derived from laboratory experiments and species' occurrences may express different aspects of the species' niche. However, there is no study systematically testing this prediction in a given taxonomic group while controlling by potential phylogenetic inertia. We estimate the thermal niches of twelve Palaearctic diving beetles species using physiological data derived from experimental analyses in order to examine the extent to which these coincided with those estimated from distribution models based on observed occurrences. We found that thermal niche estimates derived from both approaches lack general congruence, and these results were similar before and after controlling by phylogeny. The congruence between potential distributions obtained from the two different procedures was also explored, and we found again that the percentage of agreement were not very high (∼60%). We confirm that both thermal niche estimates derived from geographical and physiological data are likely to misrepresent the true range of climatic variation that these diving beetles are able to tolerate, and so these procedures could be considered as incomplete but complementary estimations of an inaccessible reality.

## Introduction

A basic aim of ecology is to understand the causes of the distribution of organisms, the niche concept being a central paradigm in approaching the problem (e.g. [1]–[2]). If the realized distribution of a species is controlled largely by environmental factors, then species distribution (SDM) or niche models may help us to disentangle the factors that set distributional limits [3]–[6]. In SDM procedures, correlational techniques are used to identify key suites of environmental conditions within which the species is present, based on environmental data from available occurrence records. Thus, predicted distributions reflect those areas in which a species is predicted to occur, based on environmental conditions in known localities [7]. However, the estimation of species environmental limits based on occurrence data is not an easy task, because even if all current populations of a given species are included in analyses, maximizing the likelihood of including environmental extremes [8], the environmental range encompassed by these limits could be smaller than the real one [6]. This is especially true if we consider those areas where species become extinct for non-environmental reasons [9], or even if dispersal limitations or biotic interactions hinder the colonization of the whole, potentially, favourable area [7], [10].

The range of species environmental limits could be alternatively estimated via experimental (mainly physiological) studies [11]–[13]. Different ecophysiological variables may facilitate identification of the constraints which prevent species occupying a wider spectrum of available conditions in nature, restricting them to certain environmental bounds beyond which the species cannot survive. In contrast to correlative models, such mechanistic or physiological models incorporate explicit relationships between environmental conditions and organismal performance, estimated independently of current distributions [14]. These sophisticated models are often too specialized and data-hungry (and typically focused on vertebrates) to be of general use in species management, especially for rarer species and/or invertebrates [15].

Among the many possible niche dimensions, thermal tolerances are frequently linked with distributional ranges (e.g. [16]–[20]), so the characterization of thermal limits would be crucially important in order to assess whether a species could successfully colonize a new site. Unfortunately, the lack of formal tests and the influence of a high number of cross-correlated and alternative factors hinder the establishment of a direct causal link between thermal limits and geographical distributions [21]–[22]. Temperature is probably the most important environmental factor influencing the performance of species, especially in ectotherms, which are the majority of species on Earth.

Although it is expectable that thermal physiology data and species' occurrences may express different aspects of the species' niche, there is no study systematically testing this prediction in a given taxonomic group while controlling by potential phylogenetic inertia. In this study we estimate the climatic niche of 12 congeneric Palaearctic species of diving beetles (and its geographical projection) following two of the most widely used approaches. We estimated thermal limits obtained from i) species distribution models based on observed occurrences and ii) thermal physiological data derived from experimental analyses in order to examine their congruence. We further aim to assess whether dispersal capacities, range attributes can explain, at least partially, the mismatches between estimates of climatic niche and potential distributions based on these two different approaches.

## Methodology

### Source of biological data

We concentrated our study on 12 species and well established sub-species from the genus *Deronectes* (family Dytiscidae) (see Table 1). This genus was selected because it is taxonomically stable with a relatively well known biology and life history, and because a previous study [13] investigated the thermal limits and dispersal abilities of these species, demonstrating that thermal physiology was the best predictor of differences in geographical range size and position.

**Table 1:** Values of thermal tolerance, size of potential distribution, geographical range and dispersal capacity for the considered species.

Species	MaxTWM	MinTCM	TR_O_	UTL	LTL	TR_PH_	DTR	PD_O_	PD_PH_	CPD	APD	DC	S Lim	N Lim	LRE
*D. algibensis* Fery & Fresneda, 1988	29.1	5.3	23.8	45.68	−3.4	49.08	25.28	340	13824	13824	2.46	1.16	36	36.5	0.5
*D. angusi* Fery & Brancucci 1990	25.5	−4.2	29.7	43.62	−7.68	51.3	21.60	6248	13728	13728	45.51	1.00	42	43.5	1.5
*D. aubei aubei* (Mulsant 1843)	29.0	−8.8	37.8	44.06	−8.34	52.4	14.60	17339	16791	18598	83.51	1.12	44	48.0	4.0
*D. bicostatus* (Schaum 1864)	34.5	−4.2	38.7	44.47	−9.43	53.9	15.20	13672	26719	26719	51.17	1.15	40	43.0	3.0
*D. depresicollis* (Rosenhauer 1856)	32.7	−7.6	40.3	45.2	−7.69	52.89	12.59	17892	19269	19269	92.85	1.11	37	38.0	1.0
*D. fairmairei* (Leprieur 1876)	39.9	−5.9	45.8	45.74	−7.39	53.13	7.33	31879	44739	44739	71.26	1.30	31	47.5	16.5
*D. hispanicus* (Rosenhauer 1856)	33.4	−7.6	41	45.57	−5.15	50.72	9.72	21768	18802	24722	64.10	1.20	36	44.5	8.5
*D. latus* (Stephens 1829)	28.4	−19.7	48.1	46.91	−9.96	56.87	8.77	35746	21222	37203	53.13	1.01	41	69.0	28.0
*D. mazzoldi* Fery & Brancucci 1990	33.3	−7.4	40.7	44.7	−6.09	50.79	10.09	11579	9423	11595	81.13	1.25	40	42.0	2.0
*D. opatrinus* (Germar 1824)	36.0	−5.8	41.8	45.63	−6.46	52.09	10.29	18614	21793	21793	85.41	1.21	36	45.5	9.5
*D. semirufus* (Germar, 1844)	29.6	−6.5	36.1	42.63	−9.06	51.69	15.59	13188	19224	19224	68.60	1.08	42.5	45.5	3.0
*D. wewalkai* Fery & Fresneda 1988	32.5	−3.6	36.1	42.83	−9.08	51.91	15.81	5049	11999	11999	42.08	1.07	40	41.0	1.0

Highest value of the maximum temperature of the warmest month (MaxTWM), lowest value of the minimum temperature of the coldest month (MinTCM) and thermal range (TR_O_) from occurrence data (°C); Upper Thermal Limit (UTL), Lower Thermal Limit (LTL) and thermal range (TR_PH_) from physiological experiments (°C); difference between both thermal ranges (DTR =  TR_PH_ – TR_O_); Number of pixels (0.2degrees) of the potential distribution using climatic data derived from occurrences (PD_O_) and physiological thermal limits (PD_PH_ ); Combined potential distribution map using both methods (CPD) and percentage of agreement between these two approaches for estimating potential distributions (APD) (see methods for details); Dispersal Capacity (DC); and Southern (S Lim) and northern (N Lim) range limits (degrees), and latitudinal range extents (LRE) for the *Deronectes* species studied.

For each of these taxa we compiled georeferenced distributional data across their entire ranges to obtain information on the general climatic conditions encompassed by the environmental extremes of their complete distribution. A total of almost 900 clean database records were obtained from 25 specialized publications, an exhaustive Iberian database (ESACIB, [23]), the GBIF database, data from the ckmap project [24], and other unpublished data including private collections. Although such data can be considered both partial and biased, due to the unavoidable lack of homogeneity in taxonomic and faunistic effort across regions, the inclusion of all these data is the best available approach to represent the climatic conditions where species occur [8]. All biological information was georeferenced at a 0.2 degree spatial resolution (cells of 100 km^2^, approximately).

### Climatic variables

Two geographically derived variables have been used as thermal-niche predictors: maximum temperature of warmest month (MaxTWM) and minimum temperature of coldest month (MinTCM). We selected MaxTWM and MinTCM for this study because they are the available variables that best express the temperature extremes in each cell. These variables allow us to obtain an estimation of the thermal niche tolerance to heat and cold from occurrence data. As this group of aquatic species is highly dependent on the existence of watercourses, we limit the potential distribution derived from these temperature values to those localities with sufficient rainfall to allow occurrence (based on the precipitation values of occupied cells). Thus, two additional variables were also considered when estimating potential distribution: precipitation of wettest month (PWM) and precipitation of driest month (PDM). All the climatic variables were obtained at the same resolution as the biological data (i.e. 0.2 degree cells) from WorldClim (version 1.3, http://www.worldclim.org; see [25]).

### Physiological data

To define species' thermal biology, we used data on upper thermal limits (UTL) and lower thermal limits (LTL) previously established for the twelve considered taxa [13]. To obtain these data all species were collected during spring and summer [13] from a single location towards the centre of each species range. All individuals were early post-teneral adults, minimizing possible confounding effects of age. The number of individuals used ranged from 28 (*Deronectes angusi*) to 92 (*D. hispanicus*). Collected individuals were transported to the laboratory in thermally insulated containers and maintained in aerated artificial pond water and fed chironomid larvae *ad libitum*. Each species was divided haphazardly into two equal groups, acclimated at 14.5 or 20.5°C respectively for 7 days before thermal tolerance experiments were conducted to determine upper and lower thermal tolerances. We used upper and lower lethal thermal limits in our analysis here because these are the most reliable and repeatable measures of thermal limits in diving beetles. These limits were assessed by means of thermal ramping experiments (for methodological details see [13], [20]).

### Assessing congruence in thermal limits

UTLs and LTLs obtained from physiological experiments were compared with those estimates of heat and cold tolerance obtained from occupied localities via linear regressions, assuming that both variables have similar random distribution errors (see [26]). Here a statistically significant relationship will suggest that the two methods of thermal niche estimation are congruent, and if the slope of the regression line is not different from unity, we also may assume that the critical thermal limits derived from the two procedures generate comparable thermal niche estimations and potential distributions.

**Figure 1:**
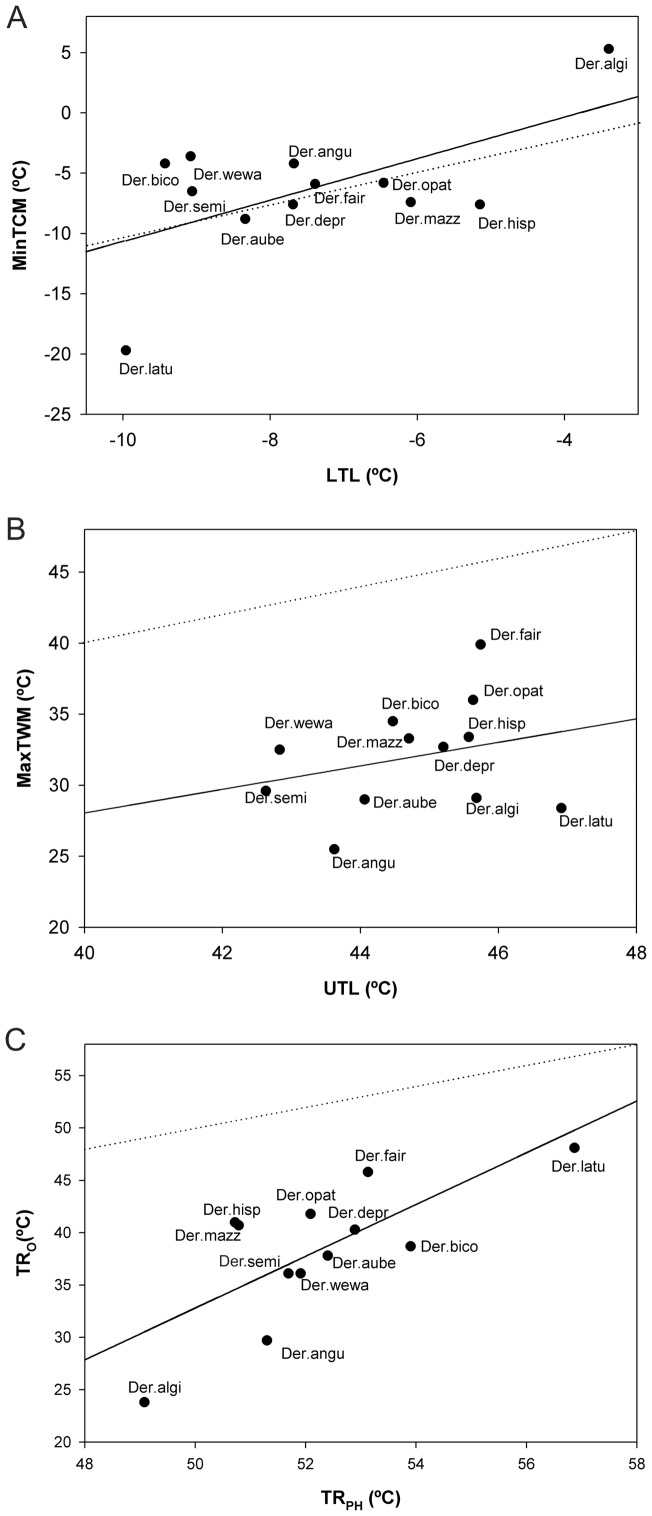
Tolerance to cold, heat and thermal range. Relationships between the tolerance to cold (A), heat (B) and thermal range (C), obtained from environmental data based on site occupancy (vertical axis) and from physiological experiments (horizontal axis). A) Highest value of the Maximum temperature of the warmest month (MaxTWM) from occurrence data, Upper Thermal Limit (UTL) from physiological experiments; B) lowest value of the minimum temperature of the coldest month (MinTCM) from occurrence data, Lower Thermal Limit (LTL) from physiological experiments; and C) thermal range (TR_O_) from occurrence data and thermal range (TR_PH_) from physiological experiments. Continuous line represents the regression line; dotted line is equality. Species names are abbreviated.

For each species we also calculated the difference between thermal limits obtained by both procedures (difference of heat limits, DHL  =  UTL-MaxTWM in occurrence localities; and difference of cold limits, DCL  =  LTL-MinTCM in occurrence localities). These values correspond to the distance of each species' thermal limit based on occurrence data from the equality line of this relationship (see Fig. 1). These deviations (DHL and DCL) can be considered as a measure of the capacity of a species to inhabit warmer or colder conditions than estimated by physiology or, alternatively, its inability to colonize *a priori* suitable mesoclimatic conditions.

The relationships between DHL/DCL and species dispersal capacities (DC) were examined using Spearman rank correlations. This approach allows us to identify whether the limitations of distributional data to estimate thermal limits are associated with differences in the dispersal capacities of species. DC values were obtained from [13] using wing length/body length ratio as a comparative measure of the relative dispersal capacity of these beetles (see also [27]).

We lastly calculated the thermal range derived from occurrence data (TR_O_  =  MaxTWM-MinTCM in occupied localities) and from physiological experiments (TR_PH_  =  UTL-LTL), using the difference between both range values (DTR  =  TR_PH_-TR_O_) as a measure of congruence in the thermal tolerances obtained from the two procedures. We assessed if these differences in thermal tolerance ranges (DTR) were associated with three general attributes characterizing species geographical ranges (northern limit of distribution, southern limit of distribution and latitudinal range extent in degrees) and also with the dispersal capacity (DC), using Spearman rank correlations.

### Potential distributions

We used multidimensional-envelopes (MDEs) to estimate the potential distribution of each species according to the conceptual and methodological guidelines proposed by Jiménez-Valverde and colleagues [6]. Considering a potential-realized distribution gradient, different modelling methods may be arranged along this gradient according to their ability to model any concept (while potential distribution refers to the places where a species could live, realized distribution refers to the places where a species actually lives). Following Jiménez-Valverde and colleagues [6], complex techniques able to use presence-absence data and parametrize the role played by contingent non-climatic factors may be more suitable to model the realized distribution than simple ones based solely in the information provided by the available presence data, which may be more appropriate to estimate the potential distribution. In this study we decided to use a multidimensional-envelope procedure (MDE) because it provides a picture close to the potential distribution (not realized one) from observed occurrences. First, we estimated for each species the climatic values prevailing in observed occupied localities, and then calculated extreme climatic values. For each species we calculated the maximum temperature of the warmest month (MaxTWM), the minimum temperature of the coldest month (MinTCM), the maximum precipitation of the wettest month (PWM), and the minimum precipitation of the driest month (PDM) for each occupied cell (0.2 degrees). These extreme values were used to derive a distributional hypothesis of areas with climatically suitable conditions (the potential distribution), assuming that these recorded occurrences are representative of the full environmental spectrum of climatic conditions in which the species may survive and reproduce. Two binary potential distribution maps were derived for each species: one using climatic data derived from occurrences (PD_O_), and the other built with the physiological thermal limits (PD_PH_) derived from physiological experiments [13], being both models restricted by the values of precipitation obtained from occurrence data. Maps derived from physiological data assume that the two climatic variables reflecting mesoclimatic conditions in each cell acts as accurate representations of physiological thermal limits. This assumption is supported by a previous comparison of PD_PH_ values against the values of all pairwise WorldClim temperature related variables. Both PD_O_ and PD_PH_ maps were overlapped to assess the spatial congruence between the potential distributions obtained by these two different procedures. Thus, we firstly overlaid both maps to obtain a combined potential distribution map (CPD), and the percentage of agreement between these two approaches was calculated as a measure of the area shared by both methods on the CPD.

In the same way as for the thermal niche, we lastly tried to investigate if the spatial congruence (or differences) in the potential distributions derived from these two procedures was associated with three general characteristics of species geographical ranges (northern and southern limit of distribution; latitudinal range extent in degrees) and also with dispersal capacity (DC), using Spearman rank correlations.

### Phylogenetic analyses

To account for potential non-independence due to shared evolutionary history, our results were feed-back in a phylogenetic framework. When significant relationships were detected with raw data, these were further explored in a phylogenetic framework based on the phylogenies for this group provided by Abellán and Ribera [28] updated with recent unpublished data. For this purpose we used the Phylogenetic Generalized Least Squares approach (PGLS; [29]) as implemented in Compare 4.6 b [30]. PGLS is a generalized case of the more widely known Independent Contrasts method. To assess the significance of the relationship we used the corMartins function of the R package ‘Ape’ [31] with the estimated value of alpha to create the correlation structure, and then fitted the linear model with the gls function (see [28 for details]). All these relationship were also significant (P<0.05) according to PGLS and are shown in Table S1.

## Results

### Estimated thermal niches

Lower thermal limits estimated from occurrence data and physiological experiments are significantly correlated (*F*_(1, 10)_ = 5.51, *P* = 0.04; Fig. 1A) whilst estimates of upper thermal limits are not (*F*_(1, 10)_ = 0.81, *P* = 0.39; Fig. 1B). The slope of the relationship for cold limits is 1.72±1.64 (±95% confidence interval), which is not significantly different from unity. However, this relationship is highly dependent on the two extreme cases (Fig. 1B), and when these are excluded the relationship is no longer significant (*F*_(1, 8)_ = 2.02, *P* = 0.19).

DHL values seem to be significantly higher than DCL ones (Wilcoxon Matched Pairs Test for dependent variables; Z = 3.06; P = 0.002; see Fig. 1). Thus, maximum temperatures of the warmest month in occupied localities do not exceed estimated physiological thermal limits (Fig. 1B), but the coldest climatic conditions in inhabited localities are nearer to lower thermal limits as estimated in the laboratory. Four species (*Deronectes aubei aubei*, *D. hispanicus*, *D. latus* and *D. mazzoldi*) occur in sites with minimum temperature values colder than their estimated mean physiological limits (Fig. 1A). DHL was significantly and negatively correlated with dispersal capacity (*rs*  = −0.678; *P* = 0.01). However, this correlation was not significant in the case of DCL (*rs*  = 0.147; *P* = 0.65).

In general, the values estimated by physiological experiments suggest higher tolerance values than those estimated from occurrence information (Wilcoxon Matched Pairs Test for dependent variables; Z = 0.02; *P*<0.01). Linear regression revealed a statistically significant relationship between TR_O_ and TR_PH_ (*F*_(1,10)_ = 11.18, *P* = 0.007) with a slope of 2.47±1.65 (±95%confidence interval) that did not differ substantially from the unity (Fig. 1C). However, as for cool limits, this relationship is also highly dependent on the two extreme cases (Fig. 1C), and when these are excluded the relationship is no longer significant (*F*_(1, 8)_ = 0.69, *P* = 0.43).

Differences in thermal tolerance ranges (DTR) estimated by the two procedures are only significantly and negatively correlated with the latitudinal range extent of species (*rs*  = −0.79; *P* = 0.002).

### Potential distributions

**Figure 2:**
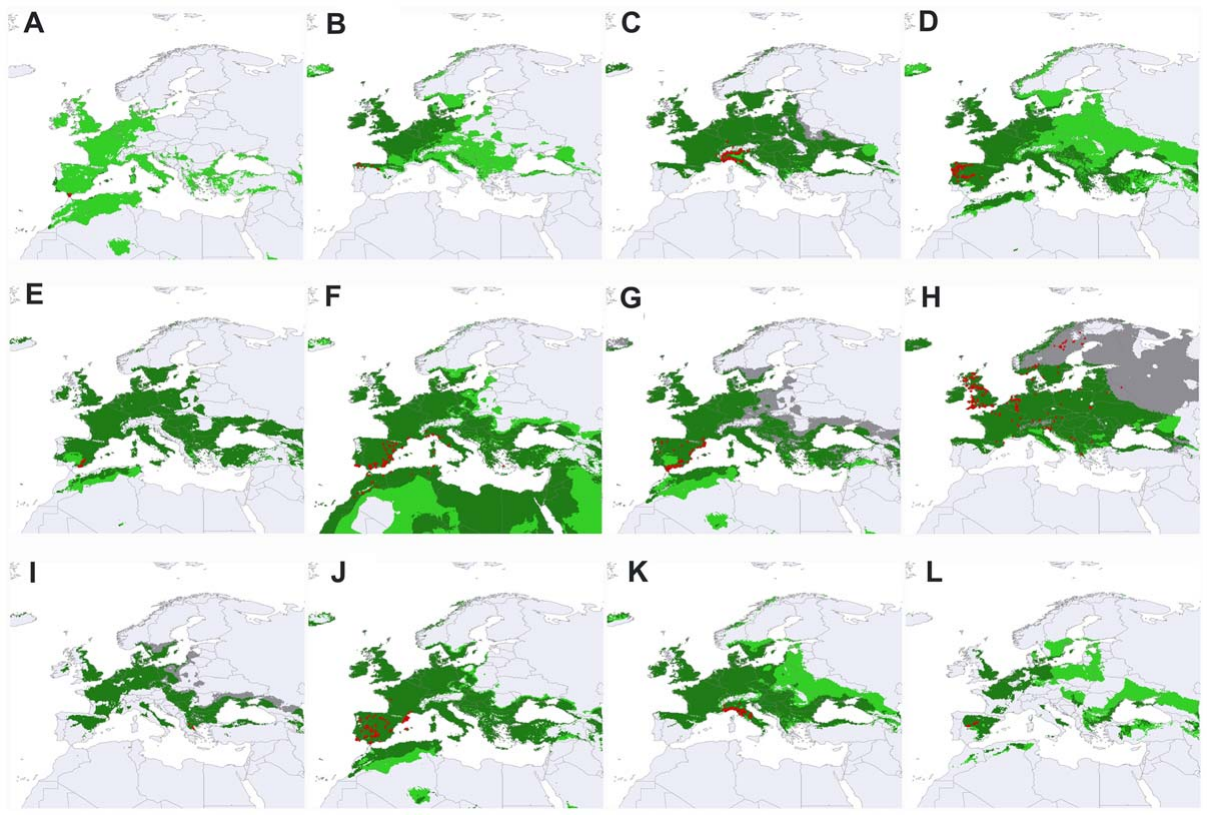
Potential distribution maps. Grey surface represents the area that is predicted as potential distribution only using climatic data from occurrences; light green surface represents the area that is predicted as potential distribution only using physiological tolerance; dark green surface represents the potential area shared by the two procedures (i.e. areas where both methods overlap). For each species, the combined potential distribution (CPD) using both methods is represented as the sum of the three colours. Red points indicate occupied localities. A: *D. algibensis*; B: *D. angusi*; C: *D. aubei aubei*; D: *D. bicostatus*; E: *D. depresicollis*; F: *D. fairmairei*; G: *D. hispanicus*; H: *D. latus*; I: *D. mazzoldi*; J: *D. opatrinus*; K: *D. semirufus*; L: *D. wewalkai*.

The differences between the potential distributions generated using the thermal limits estimated from occurrences (PD_O_) and physiological tolerances (PD_PH_) as well as the combined potential distribution map using both methods (CPD) are shown in the Figure 2.

We found a significant positive relationship between the size (number of cells) of PD_O_ and PD_PH_ potential distributions (*F*_(1, 10)_ = 7.61; P<0.05). For eight of the twelve studied species PDO values were smaller than PD_PH_ ones (see Table 1), although this difference was not statistically significant overall (Wilcoxon Matched Pairs Test for dependent variables; Z = 1.49; P = 0.136; see Fig. 2). PD_O_ and PD_PH_ represented, on average, 69.7 and 92.1% of the whole CPD, respectively. However, these percentages were not significantly different (Wilcoxon Matched Pairs Test for dependent variables; Z = 1.72; P = 0.08; see Fig. 2). The percentage of agreement between these two approaches to estimate potential distributions (APD) was calculated for each species, resulting in average 66.4±25.0 (median ± SD). These values did not statistically correlate with either dispersal capacity or with the three characteristics of species ranges.

## Discussion

Our results suggest that at least for the diving beetles considered, thermal limits and tolerances derived from geographical and physiological data showed only partial congruence, since the two procedures used to infer thermal niches are not always correlated. In the same way, their geographical projections (in the form of potential distributions) are only partially congruent (around 60%).

Lower thermal limits (LTL) as estimated from physiological data could only be partially predicted from distributional data, and several species do occur in sites with minimum temperatures that are close to, or even exceed, their physiological thermal limits (i.e. species living below reported lower thermal limits). This result may be explained considering the ecology of these insects in more detail. Firstly, although members of the group can be collected from the water during winter, some individuals may also overwinter on land, as observed in other dytiscids [32], meaning that they frequently experience sub-zero temperatures. In this sense, a flexible behavioural response may alleviate the apparent constraints of physiological tolerance limits [22], [33]–[35]. Secondly, selection of overwintering micro-spatial places with higher temperatures than the surrounding area and aggregation of individuals are common strategies to avoid exposure to potentially harmful low temperatures [36]. On the other hand, another important factor explaining this mismatch between physiological and field observations could be that physiological limits for each species were obtained using individuals collected as close as possible to the central point of their latitudinal ranges, to ensure comparable data across species [13], [37]. One of the main disadvantages of such an approach is the assumption of species homogeneity, and local adaptation and differences in the degree of phenotypic plasticity of populations could result in higher thermal tolerance, and wider predictions of potential distributions [38], [39].

The mismatch between laboratory results and field observations is especially evident in the case of the UTL since i) the upper physiological thermal limits could not be predicted from distributional data and ii) none of the species considered seem to be able to colonize regions with mesoclimatic conditions near to the upper limit of their thermal niche. In this case, species with lower dispersal abilities are also those with a larger portion of the predicted suitable warmer part of their thermal niche that has not been colonized yet, as showed by the negative correlation between dispersal capacity and the deviation of the physiological upper thermal limit (UTL) from that estimated from occurrences. The mismatch in UTL estimates could also be due to differences in the effect of other factors that prevent the establishment of stable populations when temperature is suitable but far from optimal [40]. In this regard other environmental variables such as precipitation or non-climatic factors could make physiological limits to heat less evident predictors of actual distribution [41]. It is worth noting that although these are aquatic animals, and maximum and minimum water temperatures in streams are generally less extreme than those in air, these beetles spend part of their life cycle on land. The pupal stage and the early adult stages of dytiscids, for example, occur on land in small burrows beside the water [32] and individuals will be exposed to greater temperature fluctuations than when they are submerged. In *Deronectes*, this stage takes place mainly in spring and early summer, when air temperatures are relatively high.

Thermal tolerance ranges estimated by the two methods are not highly congruent. These differences in tolerance values are related to geographical range size, the most geographically restricted species showing greater differences in thermal ranges estimated by the two methods. Narrowly endemic taxa may be more important from a conservation perspective [42], and our results suggest that caution is especially recommended when estimating thermal niches (and their geographical projections) using information from occurrence data with such species. In this sense, correlative species distribution models may fail to unveil thermal niches (specially for narrow endemic taxa) because the area currently occupied by a species can only provide partial environmental information on the full set of abiotic conditions under which the species can survive and reproduce [6], [8], [10], [43]. In the case of *Deronectes* species, the warmer portion of the thermal niche could be misrepresented if occurrence data alone are taken into account. This methodological problem appears when the cause of these restricted ranges is not limited by establishment ability, as determined by fundamental niche breath, but by limited dispersal capacity, competitive exclusion or other non-climatic factors [4], [6]. Since a number of historical and ecological processes may determine geographical range size [41], the estimation of the potential distribution of a species based only on occurrence information could be biased by these same processes. Physiologically suitable areas are more difficult to distinguish because they need a reliable climatic variable as a surrogate. Ultimately those areas that do not appear physiologically appropriate from geography (distributional data) should have lower suitability values because they are probably determined by unknown factors preventing colonization. This casts doubt on the possibility of understanding what restricts the occurrence of taxa through correlational approaches alone.

On the other hand, experimental approaches alone may also fail to represent the thermal niche of a species (especially in the case of widely distributed taxa) for different reasons [44]. Distinct populations may possess different thermal tolerances [45], acclimation and plasticity may also alter inferred thermal niche values [22], or unknown environmental factors may buffer exposure to lethal temperatures [46]. Potential distribution models based on physiological data from individuals from a single population could also misrepresent the species' true potential distribution range since they do not take into account inter-population variability or different behavioural adaptations which may facilitate acclimation to extreme temperatures [22], [47], [48].

The degree of congruence between approaches applied to estimate potential distributions was unrelated to estimates of relative dispersal capacity, or the size and position of species ranges. It seems therefore that, for the species here considered, the relative dispersal capacity of these species is not a good predictor of both species latitudinal range extent [13] or the degree of congruence between the two approaches to estimate potential distributions. This situation could be explained only if non-climatic factors (e.g. competitive exclusion) are shaping the distributional ranges of taxa far from their climatic equilibrium [49]. The limited agreement between the two approaches employed here to estimate potential ranges suggest highlighting the importance of taking multiple methodologies into account if we are to gain more accurate estimates of the potential distribution of individual species.

### Concluding remarks

Our results suggest that thermal limits and tolerances derived from geographical and physiological data may lack general congruence. In this sense, thermal niches derived from physiological experiments and geographical data may be considered incomplete but complementary estimations (e.g. [14,44,50,51) of an inaccessible reality. Individual procedures to estimate species fundamental niches are likely to misrepresent the true range of climatic variation that taxa are able to tolerate.

Although our study is based on a single clade of beetles, there is no reason to suspect that such findings do not generalise, particularly for species with complex life-cycles such as diving beetles, which are exposed to a wide variety of microclimates during their ontogeny. As a consequence we suggest that procedures which rely on estimations of potential distributional ranges, such as the identification of additional survey sites [52], estimations of niche conservatism [53], [54], assessments of species range shifts under climate change [55]–[56], identification of important areas for conservation [57] or estimations of invasion risk [58], [59] might reduce inherent uncertainty by integrating distributional and physiological data.

## Supporting Information

Table S1 **Relationship once controlled for phylogenetic relatedness.** Results of the significant relationship with raw data once controlled for phylogenetic relatedness (see text for details). LTL: Lower Thermal Limit; MinTCM: lowest value of the minimum temperature of the coldest month; DHL: difference of heat limits obtained by both procedures, DC: Dispersal Capacity; TR_O_: thermal range from occurrence data (°C); TR_PH_: thermal range from physiological experiments (°C); DTR: difference between thermal ranges obtained by both procedures; LRE: latitudinal range extent; PD_O_: Number of pixels (0.2degrees) of the potential distribution using climatic data derived from occurrences and (PD_PH_ ) physiological thermal limits.
(DOC)
