# Supplementary material for: Assessing the Congruence of Thermal Niche Estimations Derived from Distribution and Physiological Data. A Test Using Diving Beetles
Source: PLoS One. 2012 Oct 25;7(10):e48163. doi: 10.1371/journal.pone.0048163 (PMC3485027; doi:10.1371/journal.pone.0048163)
Supplement: Table S1 — Relationship once controlled for phylogenetic relatedness. Results of the significant relationship with raw data once controlled for phylogenetic relatedness (see text for details). LTL: Lower Thermal Limit; MinTCM: lowest value of the minimum temperature of the coldest month; DHL: difference of heat limits obtained by both procedures, DC: Dispersal Capacity; TRO: thermal range from occurrence data (°C); TRPH: thermal range from physiological experiments (°C); DTR: difference between thermal ranges obtained by both procedures; LRE: latitudinal range extent; PDO: Number of pixels (0.2degrees) of the potential distribution using climatic data derived from occurrences and (PDPH ) physiological thermal limits. (DOC) [file pone.0048163.s001.doc]

**Table S1.** **Relationship once controlled for phylogenetic relatedness.**

Results of the significant relationship with raw data once controlled for phylogenetic relatedness (see text for details). LTL: Lower Thermal Limit; MinTCM: lowest value of the minimum temperature of the coldest month; DHL: difference of heat limits obtained by both procedures, DC: Dispersal Capacity; TR_O_: thermal range from occurrence data (ºC); TR_PH_: thermal range from physiological experiments (ºC); DTR: difference between thermal ranges obtained by both procedures; LRE: latitudinal range extent; PD_O_: Number of pixels (0.2degrees) of the potential distribution using climatic data derived from occurrences and (PD_PH_ ) physiological thermal limits.

|  | α | *r^2^* | *P* |
| --- | --- | --- | --- |
| LTL and MinTCM | 15.5 | 0.581 | <0.05 |
| DHL and DC | 15.5 | -0.751 | <0.05 |
| TR_O_ and TR_PH_ | 1.24 | 0.967 | <0.05 |
| DTR and LRE | 1.3 | -0.931 | <0.05 |
| PD_O_ and PD_PH_ | 15.5 | 0.631 | <0.05 |
